# Early changes in myocyte contractility and cardiac function in streptozotocin-induced type 1 diabetes in rats

**DOI:** 10.1371/journal.pone.0237305

**Published:** 2020-08-21

**Authors:** Gustavo S. Marchini, Ismar N. Cestari, Vera M. C. Salemi, Maria Claudia Irigoyen, Alexandre Arnold, Adélia Kakoi, Camila Rocon, Vera D. Aiello, Idágene A. Cestari

**Affiliations:** 1 Biomedical Engineering Graduate Progam, University of São Paulo Polytechnic School, São Paulo, Brazil; 2 Heart Institute-InCor University of São Paulo Medical School, São Paulo, Brazil; Scuola Superiore Sant'Anna, ITALY

## Abstract

Diabetes can elicit direct deleterious effects on the myocardium, independent of coronary artery disease or hypertension. These cardiac disturbances are termed diabetic cardiomyopathy showing increased risk of heart failure with or without reduced ejection fraction. Presently, there is no specific treatment for this type of cardiomyopathy and in the case of type I diabetes, it may start in early childhood independent of glycemic control. We hypothesized that alterations in isolated myocyte contractility and cardiac function are present in the early stages of experimental diabetes in rats before overt changes in myocardium structure occur. Diabetes was induced by single-dose injection of streptozotocin (STZ) in rats with data collected from control and diabetic animals 3 weeks after injection. Left ventricle myocyte contractility was measured by single-cell length variation under electrical stimulation. Cardiac function and morphology were studied by high-resolution echocardiography with pulsed-wave tissue Doppler imaging (TDI) measurements and three-lead surface electrocardiogram. Triglycerides, cholesterol and liver enzyme levels were measured from plasma samples obtained from both groups. Myocardial collagen content and perivascular fibrosis of atria and ventricle were studied by histological analysis after picrosirius red staining. Diabetes resulted in altered contractility of isolated cardiac myocytes with increased contraction and relaxation time intervals. Echocardiography showed left atrium dilation, increased end-diastolic LV and posterior wall thickness, with reduced longitudinal systolic peak velocity (S’) of the septum mitral annulus at the apical four-chamber view obtained by TDI. Triglycerides, aspartate aminotransferase and alkaline phosphatase were elevated in diabetic animals. Intertitial collagen content was higher in atria of both groups and did not differ among control and diabetic animals. Perivascular intramyocardial arterioles collagen did not differ between groups. These results suggest that alterations in cardiac function are present in the early phase in this model of diabetes type 1 and occur before overt changes in myocardium structure appear as evaluated by intersticial collagen deposition and perivascular fibrosis of intramyocardial arterioles.

## Introduction

Diabetes mellitus (DM) is among the leading causes of death globally and is considered a major risk factor for cardiovascular disease. Data from the International Diabetes Federation indicate that 425 million people worldwide have DM and this number may rise to 629 million until 2045 [1]. DM is a major risk factor for cardiovascular disease [2, 3] and patients with DM may suffer from myocardial disease unrelated to other cardiovascular conditions, called diabetic cardiomyopathy (DCM) [4]. DCM results from complex interactions between metabolic abnormalities arising from hyperglycemia and insulin resistance that accompany DM as well other signaling and cellular alterations [5–7]. Experimental DM studies have demonstrated alterations in sarcoplasmic reticulum and cytosolic Ca^2+^ handling with decrease in diastolic and systolic contractile function and diminished cardiomyocyte contraction [8, 9]. Passive stiffening of permeabilized cardiomyocyte has been shown to occur in DM, being related to titin phosphorylation and insulin signaling [10].

Streptozotocin (STZ) injection is one of the most frequently used rodent models of DM type 1 (T1DM) and other pathophysiological changes occurring in the myocardium with DCM [11, 12]. Single low dose injection of STZ avoids overt toxicity, produces progressive β-cell damage, local inflammation, insulitis and cardiac dysfunction [13]. The single low dose model is capable of inducing hyperglycemia associated with a state of hypersensitivity to insulin. This model is considered a convenient platform for the study mitochondrial mechanisms of β-cell glucotoxicity, reproducing some of the metabolic changes occurring in T1DM [14]. It raises circulating free fatty acids and cytokines, thus exposing the heart to the relevant signals present in the diabetic milieu [15]. Additionally, there is evidence that treatment with insulin in these conditions may partially reverse some of these changes [16, 17].

Increasing evidence has demonstrated that T1DM consequences are already present in pediatric patients, even with strict glycemic control and therapy [18, 19]. However, most studies using the STZ T1DM model are performed after 8 or more weeks of STZ injection when the presence of pathophysiological signs of DM are well stablished. Early stages in experimental T1DM are rarely investigated, in part because of a higher attention to the chronic aspects of DM in humans. Investigation of the early phase may reveal unsuspected pathophysiological mechanisms already in course, which may point to new or promising therapeutic strategies.

Contractile abnormalities of isolated cardiac myocytes (CM) have been reported in later phases of experimental DM and, similar to other findings, mostly between eight and twelve weeks after STZ injection [20–22]. However, little is known about contractility changes of CM during the early stages of DM and their relation to other DM markers. The aim of this study was to investigate the presence of myocardial alterations in the early stage of the of experimental T1DM, with all results obtained after 3 weeks of STZ injection and compared to control animals of the same age. Contractility alterations were quantified in CM isolated from left ventricle (LV) and cardiac function was evaluated in these diabetic rats by high-resolution echocardiography, electrocardiography (ECG) and complemented with biochemical measurements. Collagen content and perivascular fibrosis was measured histologically using picrosirius red staining of atrium and ventricle from both groups.

## Methods

All experiments were in accordance with the Guidelines for Ethical Care of Experimental Animals from the International Animal Care and Use Committee. This study was approved by the Ethical Committee for Animal Use of the University of São Paulo Medical School (Protocol Number: 061/15). A total of 29 rats (250–300 g, 8 weeks of age) were randomly divided into two groups: age-matched control (C, n = 12) and diabetic (D, n = 17) animals. High-resolution echocardiography, ECG and biochemical analysis of liver enzyme markers, triglycerides and cholesterol were performed in live control and diabetic animals. Myocyte isolation for single-cell contractility measurements and histological analysis were performed in excised hearts.

### STZ injection and blood glucose level measurement

T1DM was modeled with a single injection of STZ (50 mg/kg, Sigma-Aldrich, St. Louis, MO, USA) dissolved in citrate buffer (0.01 M, pH 4.5) as previously described [23]. Glucose blood levels and body weights were measured after a 4-hour fasting period. Glucose levels were measured just before induction and every 7 days by tail prick using a portable glucose meter (Accu-Chek, Roche, Basel, Switzerland). STZ-injected rats with blood glucose concentration ≥ 180 mg/dl 7 days after induction were included in the diabetic group.

### High-resolution echocardiography

Three weeks after induction of DM, high-resolution transthoracic echocardiography was performed by an experienced ultrasonographer in diabetic and age-matched control rats using a VEVO 2100 system (Visual Sonics, Toronto, ON, Canada) equipped with a 40 MHz transducer. Animals were anesthetized with isoflurane (5% on O_2_ flow) and placed in the dorsal decubitus position. Using 2-D guided M-mode imaging, the following parameters were obtained from short-axis view at the level of the papillary muscles: left ventricular internal diameter in end-systole (LVIDs), left ventricular internal diameter in end-diastole (LVIDd), interventricular septum thickness in systole (IVSTs) and diastole (IVSTd), posterior wall thickness in systole (PWTs) and in diastole (PWTd) [24]. Short axis view at the level of the aortic root/left atrium was used to measure left atrial dimension (LAD) in diastole. Left ventricular mass was calculated as previously described [25] and ejection fraction (EF) was calculated by the Teicholz method [26]. Fractional shortening (FS) was calculated by the following formula: (LVIDd—LVIDs)/ LVIDd. The LV dimensions including LVID, IVST, PWT and LV mass were normalized to body weight in grams and results are presented in absolute values.

Transmitral flow pulsed-wave Doppler echocardiography was used to evaluate global diastolic function, with the sample volume placed at the tips of the mitral leaflets in the apical four-chamber view [27]. The following parameters were obtained: peak velocity of early (E) and late (A) diastolic mitral waves, E/A ratio and isovolumic relaxation time (IVRT). Using tissue Doppler imaging (TDI), with the sample volume placed at the septal side of the mitral annulus, in the apical four-chamber view, early (E’), late (A’) diastolic and systolic (S′) peak velocities were measured and E’/A’ and E/E’ ratios were calculated.

### Electrocardiography

Three weeks after induction of DM, electrocardiography was performed in diabetic and age-matched control rats using a differential biological potential amplifier (Bio Amp, ADInstruments, Dunedin, New Zealand). The animals were placed individually in an induction chamber and anesthesia was induced with 5% isoflurane delivered in 100% oxygen until loss of righting reflex. During recording, the isoflurane concentration was varied in order to maintain heart rate within the normal physiological range for Wistar rats (250–450 bpm) [28]. A three-lead surface ECG was recorded with the electrodes positioned in lead II and sampled at 1-kHz. Measurements of RR interval, PR interval, P wave duration, QRS interval and QT interval were performed in 20 consecutive beats (LabChart software, version 7.3.1, ADInstruments). QT interval was corrected to eliminate its dependence with heart rate by the following formula [29]: QTc = QT/(RR/f)^1/2^; f = 150 ms.

### Isolation of cardiac myocytes

CM were isolated from diabetic rats 3 weeks after STZ injection and from age-matched control rats. In brief, rats were heparinized (5000 U/kg), anesthetized with a single intraperitoneal injection of sodium thiopental (50 mg/kg) and, after confirming deep anesthesia, euthanized by cervical dislocation. The heart was excised and placed in ice-cold oxygenated buffer solution containing (in mmol/l): 134 NaCl, 4.0 KCl, 1.2 NaH_2_PO_4_, 10 HEPES, 0.5 MgSO_4_, 1.25 CaCl_2_ and 11 D-glucose (pH 7.4 adjusted with NaOH) [30]. The heart was placed on a Langendorff system and perfused under a constant flow of 6 ml/min at 37°C. The perfusion solution was switched to a nominally Ca^2+^-free solution for 4 minutes followed by a digestion step with added collagenase type II (100 U/ml; Worthington Biochemical, Lakewood, NJ, USA) and 20 μM Ca^2+^ until the heart became pale and flaccid (20–30 min). The ventricles were separated from the heart, cut into small pieces and gently minced in 50 μM Ca^2+^ and 1% BSA (Sigma, St Louis, MO, USA) solution. Following filtration through a 200 μm nylon mesh and sedimentation, the cell pellet was washed in buffer solution with increasing Ca^2+^ concentration of 0.1, 0.2, 0.5 and 1 mM.

### Measurement of myocyte shortening

**Fig 1:**
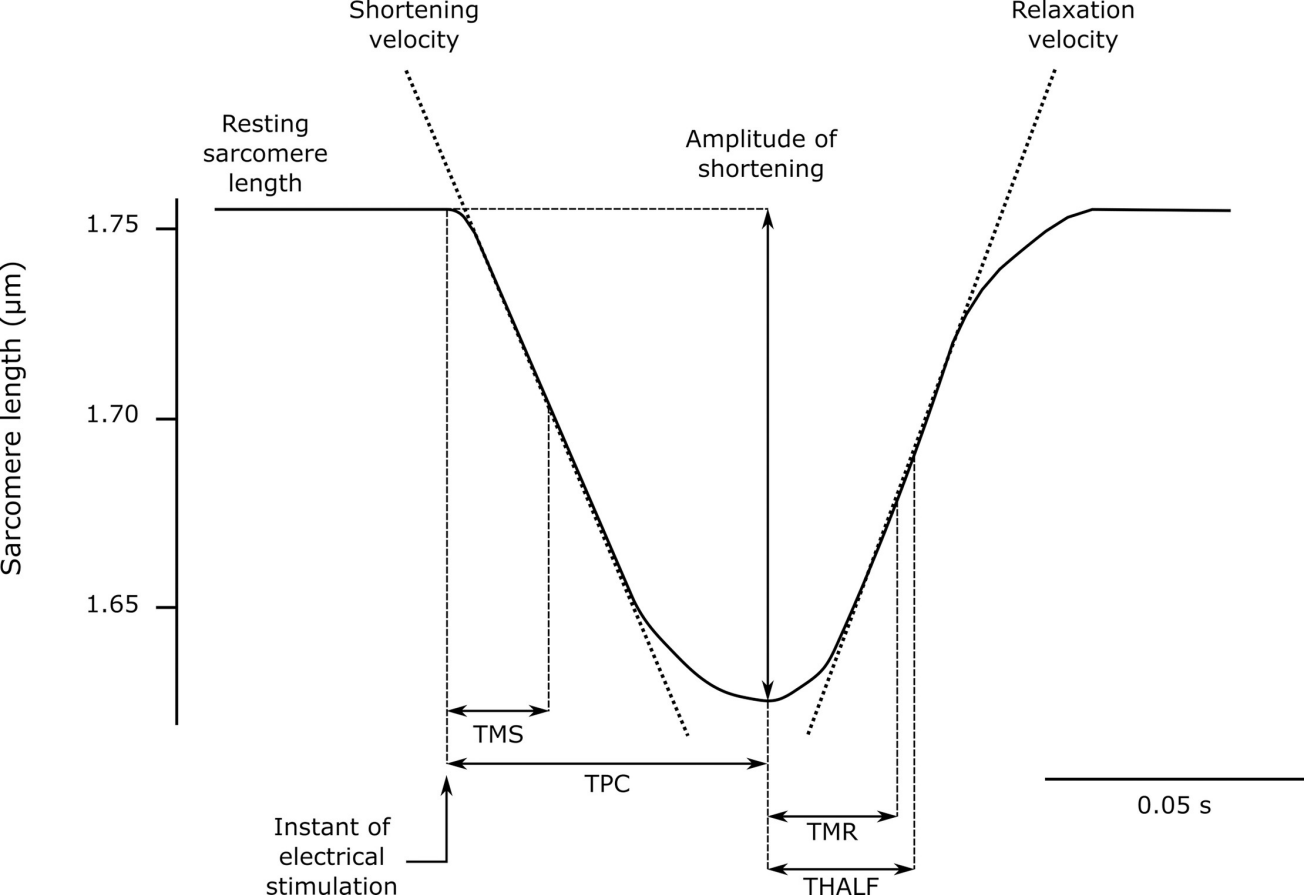
Parameters measured in the sarcomere shortening signal. TMS: time interval to reach the instant of maximal shortening velocity; TPC: time interval to reach the peak of contraction; TMR: time interval to reach the instant of maximal relaxation velocity; THALF: time interval to reach 50% resting sarcomere length.

Cardiac myocytes were placed in a perfusion chamber mounted on the stage of an inverted microscope (Eclipse TS100; Nikon, Tokyo, Japan) equipped with an analog camera (Myocam; IonOptix, Milton, MA, USA). Only rod-shaped myocytes with clear edges and without spontaneous contractions were selected for analysis. Cells were maintained at 37°C and field stimulated (MyoPacer; IonOptix) with 5 ms bipolar pulses at 1 Hz frequency using platinum electrodes placed on the opposite sides of the chamber. Contraction signals of load-free myocytes were recorded with the help of a commercial software (IonWizard; IonOptix) and the sarcomere striation pattern used to calculate changes in sarcomere spacing using a fast Fourier transform algorithm (Sarclen Algorithm; IonOptix). The following parameters were obtained (Fig 1): resting sarcomere length; amplitude of shortening (expressed as % of resting sarcomere length); shortening and relaxation velocities and time intervals to reach the peak of contraction (TPC); maximal shortening velocity (TMS); maximal relaxation velocity (TMR) and 50% resting sarcomere length (THALF). The parameters were calculated averaging at least 5 consecutive contractions.

### Biochemical measurements

Blood plasma samples were collected through the abdominal vena cava and used for biochemical analyses. Spectrophotometric measurements (BS-120 Mindray) of triglycerides (Dialab D96385b Kit), cholesterol (Dialab D96112b kit), aminotransferase and aspartate aminotransferase (Dialab D96613b kit), alkaline phosphatase (Dialab D956664 kit) and gamma glutamyl transferase (Dialab D966606b kit) were performed.

### Histopathology

After euthanasia, cardiac samples were collected, fixed in 10% neutral buffered formalin and paraffin-embedded. After paraffin removal, samples were hydrated and tissue sections cut at 3 μm were stained with Harris hematoxylin for 5 minutes with picroscirius red in saturated aqueous picric acid for 1 hour, washed for 10 minutes in HCL solution and dehydrated. Prepared sections were observed under microscopy (Leica Application suite). Magnification was set up at ×400. Micrographs were used to calculate the collagen content in the myocardial interstitium using the Image J software as the percentage of red-stained area in the section. An average of 15–20 images from each animal from each group was analyzed.

To evaluate the perivascular fibrosis of intramyocardial arterioles [31] of the ventricles, arterioles with a diameter between 100–200 μm were chosen. For each image of the arteriole, a region of interest was delimited around it to exclude areas of interstitial collagen which were not related to the arteriole. Perivascular fibrosis was determined by the ratio between the area occupied by collagen stained with picro-sirius within the region of interest and the area of the lumen [32]. The software Image J [33] was used to calculate the area occupied by the collagen and to mark the regions of interest, and the software Zen (Zeiss, Germany) to calculate the area of the lumen. The images were obtained with a 20x magnifying objective (Eclipse TE 300, Nikon, USA) with a digital camera (AxioCam, Zeiss, Germany).

### Statistical analysis

The Shapiro-Wilk test was used to evaluate normality. Data were expressed as mean ± S.D. for data with normal distribution and median and interquartile range (IQR) for data asymmetrically distributed. The comparison between groups was performed using the Student's t test for parametric variables and the Mann–Whitney for nonparametric variables. For all analyses *P* < 0.05 was considered significant. Statistical analysis was performed using SigmaPlot 11.0 (Systat Software Inc., San Jose, CA, EUA).

## Results

### General characteristics

After receiving STZ, animals showed the symptoms of hyperglycemia, such as polyuria, polydipsia and a loss in body weight generally observed in this disease model. Three weeks after induction of DM, diabetic rats displayed lower body weight (294 ± 34 g vs 342 ± 36 g, *P* < 0.01) and increased blood glucose (502 ± 72 vs 106 ± 13 mg/dl, *P* < 0.001) compared to age-matched controls.

### Echocardiography

**Fig 2:**
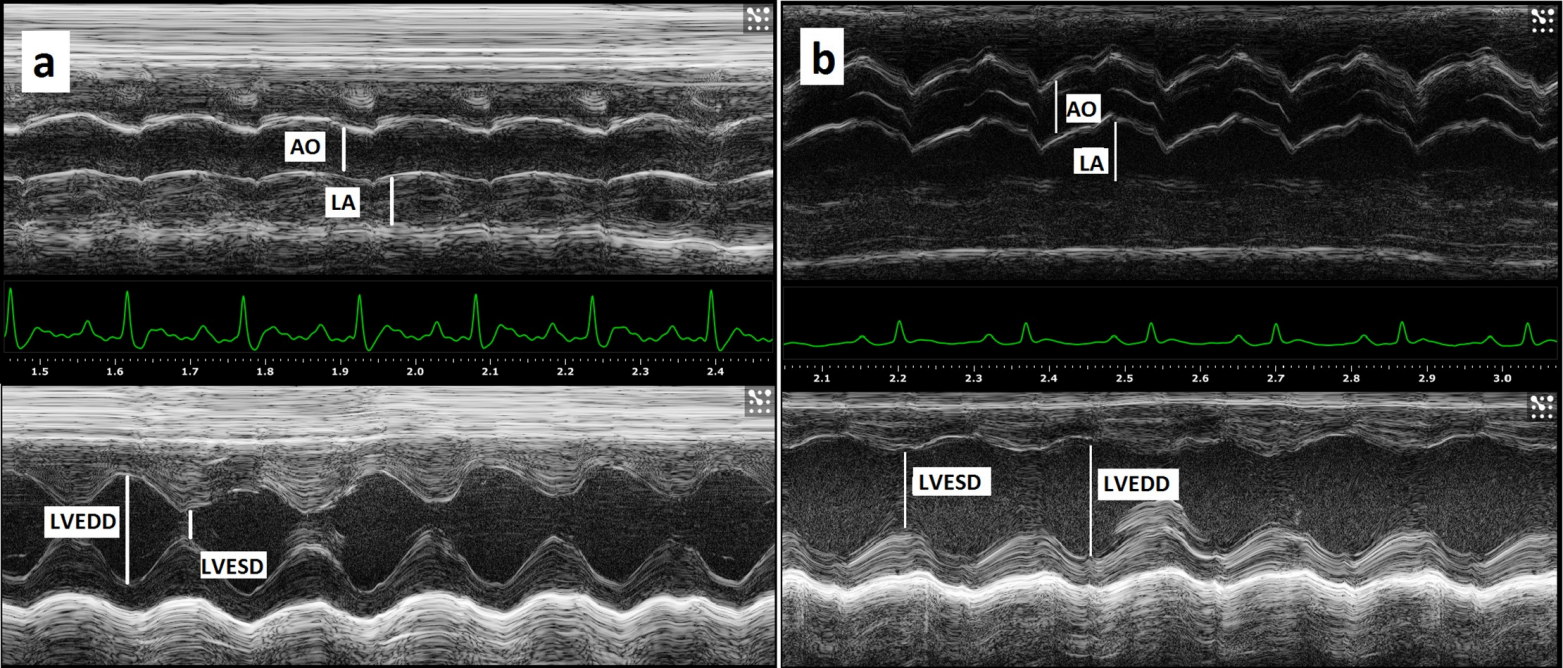
Typical echocardiogram obtained from control and diabetic rats. Bidimensional M-mode echocardiogram from a control (a) and diabetic (b) rats showing the aorta (AO) and left atrium (LA) on top and at the bottom the left ventricular cavity over the cardiac cycle during systole (left ventricle end-systolic dimension, LVESD) and diastole (left ventricle end-diastolic dimension, LVEDD) with corresponding ECG tracings.

Fig 2 shows typical echocardiogram obtained from control and diabetic rats from M-mode echocardiographic data, normalized to body weight, obtained from control (A) and diabetic (B) animals 3 weeks after diabetes induction with STZ. Left atrial diameter (LAD), LV internal diameter during diastole (LVIDd) and posterior wall thickness during systole (PWTs) were increased in diabetic animals (*P* < 0.04, Table 1).

**Table 1:** M-mode echocardiographic data obtained from control and diabetic groups.

Parameters	Control group (n = 4)	Diabetic group (n = 6)
**LAD [mm/kg]**	**9.31 ± 0.47**	14.79 ± 2.49*
LVIDs [mm/kg]	12.32 ± 2.99	15.51 ± 2.54
**LVIDd [mm/kg]**	**19.25 ± 1.31**	24.45 ± 4.15*
**PWTs [mm/kg]**	**5.25 ± 0.52**	7.28 ± 1.14*
PWTd [mm/kg]	3.85 ± 0.27	4.73 ± 1.06
LV mass [g/kg]	1.69 ± 0.16	2.18 ± 0.59
EF [%]	63.53 ± 14.75	64.51 ± 2.01
FS [%]	36.51 ± 11.42	36.44 ± 1.70

Data are expressed as mean ± standard deviation. Morphological parameters include: LAD (Left atrial dimension); LVIDs (Left ventricular internal diameter in systole); LVIDd (Left ventricular internal diameter in diastole); PWTs (Posterior wall thickness in systole); PWTd (Posterior wall thickness in diastole); EF: Ejection fraction; FS: Fractional shortening; BW: body weight.

*P < 0.04.

Table 2 shows data obtained by pulsed-wave Doppler and pulsed-wave TDI from control and diabetic groups. IVRT was corrected for heart rate by dividing the absolute value by the square root of cardiac cycle length.

**Table 2:** Pulsed-wave doppler and pulsed-wave doppler tissue imaging measured in the control and diabetic groups.

Parameters	Control group (n = 4)	Diabetic group (n = 6)
IVRTc	1.87 ± 0.70	2.15 ± 0.30
A [mm/s]	385.42 ± 159.69	446.35 ± 145.07
E [mm/s]	726.23 ± 101.00	633.27 ± 169.94
A [mm/s]	385.42 ± 159.69	446.35 ± 145.07
E/A	2.19 ± 1.00	1.54 ± 0.60
E’ [mm/s]	61.23 ± 16.72	57.41 ± 17.97
A’ [mm/s]	51.52 ± 3.38	50.61 ± 16.03
E’ [mm/s]	61.23 ± 16.72	57.41 ± 17.97
E/E’	12.61 ± 4.35	12.53 ± 7.14
**S’ [mm/s]**	**42.35 ± 1.91**	35.98 ± 5.47*

Data are expressed as mean ± standard deviation. IVRTc: Isovolumic relaxation time corrected by heart rate; A: peak velocity of late diastolic mitral inflow; E: peak velocity of early diastolic mitral inflow; S’: systolic peak velocity.

*P < 0.02.

### Electrocardiography

Table 3 shows the parameters obtained by electrocardiography 3 weeks after induction of DM. The RR interval was prolonged, heart rate was lower and P wave duration was increased in diabetic animals (*P* < 0.005). No differences were found in RR, PR, QRS and QT or QTc intervals.

**Table 3:** Values of electrocardiography in control and diabetic animals.

Parameters	Control group (n = 9)	Diabetic group (n = 14)
**RR Interval [ms]**	**163.2 ± 9.7**	187.5 ± 15.9*
**Heart rate [bpm]**	**368.7 ± 22.1**	322.1 ± 27.2*
PR Interval [ms]	52.7 ± 4.9	51.4 ± 3.7
**P wave duration [ms]**	**8.4 ± 2.6**	11.9 ± 4.0*
QRS Interval [ms]	12.2 ± 3.7	13.7 ± 3.0
QT Interval [ms]	42.8 ± 19.8	50.2 ± 17.6
QTc Interval [ms]	40.8 ± 18.0	45.1 ± 15.7

QTc = QT/(RR/f)^1/2^; f = 150 ms. Data are expressed as mean ± S.D.

*P < 0.03.

### Myocyte shortening

**Fig 3:**
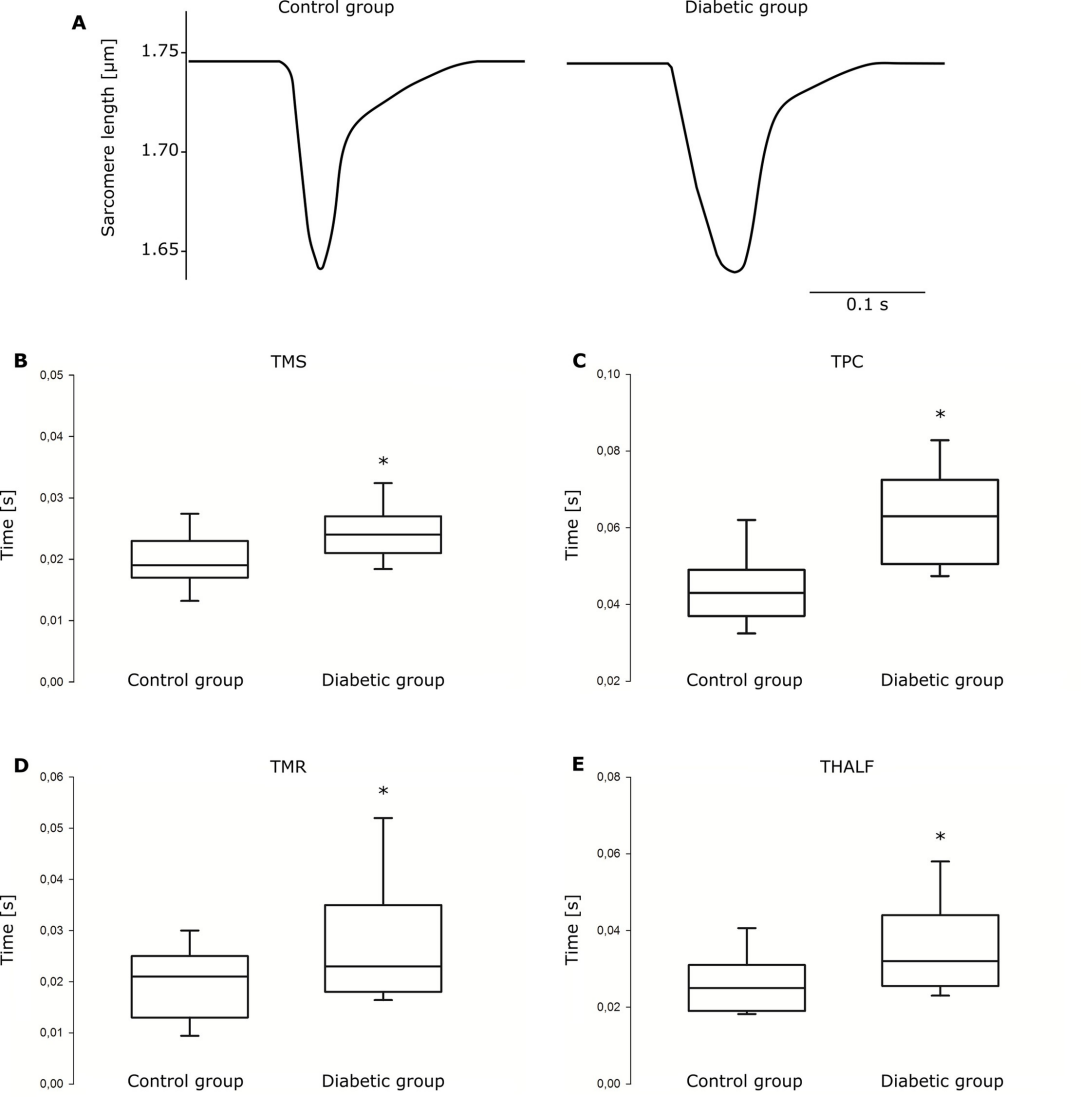
Sarcomere shortening in isolated cardiac myocytes. (A) Representative examples of sarcomere shortening in cardiac myocytes from control and diabetic rat. In the box-and-whiskers plot, the median (line), inter-quartile range (box) and 5^**th**^ and 95^**th**^ percentile (whiskers) of (B) time interval to reach the maximal shortening velocity (TMS), (C) time interval to reach the peak of contraction (TPC), (D) time interval to reach the maximal relaxation velocity (TMR) and (E) time interval to reach the 50% resting sarcomere length (THALF). n = 31 cells from 5 hearts for the control group and n = 33 cells from 8 hearts for the diabetic group. *P < 0.001.

Cells were isolated from LV of control (5 hearts, 31 cells) and diabetic (8 hearts, 33 cells) animals. The average diastolic sarcomere length was 1.75 μm in both groups. Representative records of sarcomere shortening in isolated cardiac myocytes from rat hearts of age-matched control and diabetic animals are shown in Fig 3A. The time intervals TMS, TPC, TMR and THALF were all prolonged (*P* < 0.001) in diabetic animals compared to controls (Fig 3B–3E). Shortening and relaxation velocities and amplitude of shortening were not altered in isolated cardiac myocytes from STZ-treated rats compared to control animals.

### Metabolic evaluations

Our data show elevated triglycerides in diabetic rats compared to controls and similar cholesterol levels. Enzymatic changes were found in diabetic animals, which showed increased aspartate aminotransferase and alkaline phosphatase as shown in Table 4.

**Table 4:** Values of biochemical measurements in control and diabetic animals.

Parameters	Control group (n = 4)	Diabetic group (n = 6)
Triglycerides [mg/dL]	124 (112–142)	215 (194–331)*
AST [U/L]	71 (57–79)	107 (92–242)*
ALP [U/L]	214 ± 37	599 ± 147*

AST: aspartate aminotransferase; ALP: alkaline phosphatase. Data were expressed as mean ± S.D.

*P < 0.02.

Cholesterol (77 ± 9 vs 63 ± 12 mg/dL), alanine aminotransferase (ALT, 196 ± 127 vs 47 ± 6 U/L) and gamma glutamyl transferase (GGT, 6 ± 2 vs 4 ± 2 U/L) were not different in diabetes and controls, respectively.

### Determination of myocardium fibrosis

**Fig 4:**
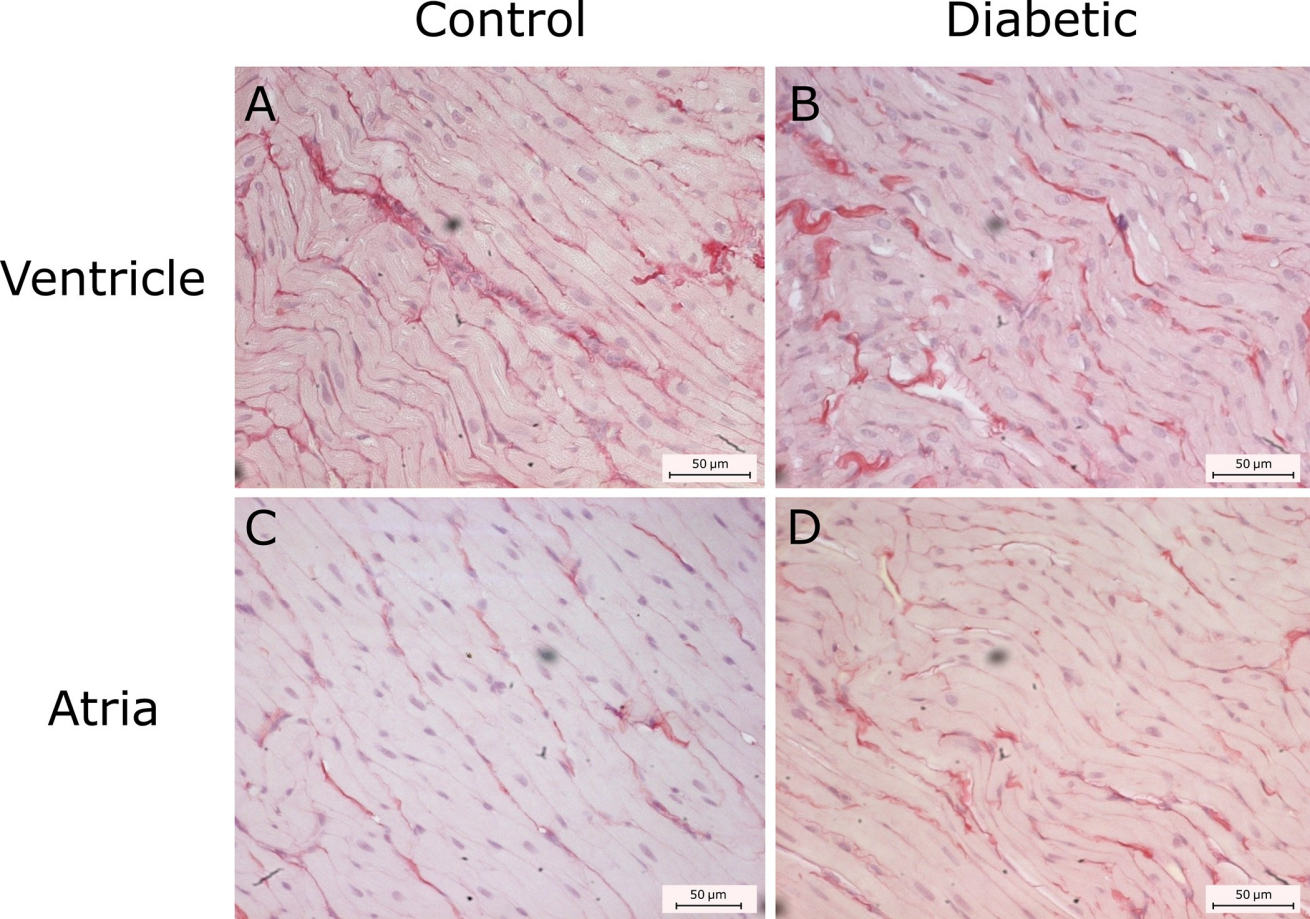
Determination of interstitial fibrosis. Interstitial fibrosis was quantified in control and diabetic rats in ventricle (A and B respectively) and atria (C and D, respectively). No changes were observed between groups both for the ventricles (*P* = 0.17) and atria (*P* = 0.42). Collagen in atrium was higher than that of ventricles in both control (*P* = 0.03) and diabetic animals (*P* = 0.01).

Collagen content was examined in the ventricular myocardial interstitium in the control group (n = 4) and in the diabetic animals (n = 5) (2.42 ± 0.43 and 2.78 ± 0.33; *P* = 0.17) and atria (4.91 ± 0.89 and 5.4 ± 0.99; *P* = 0.42) respectively. No differences were observed between groups regarding the area fraction of collagen (P = 0.36). Representative images are shown in Fig 4.

**Fig 5:**
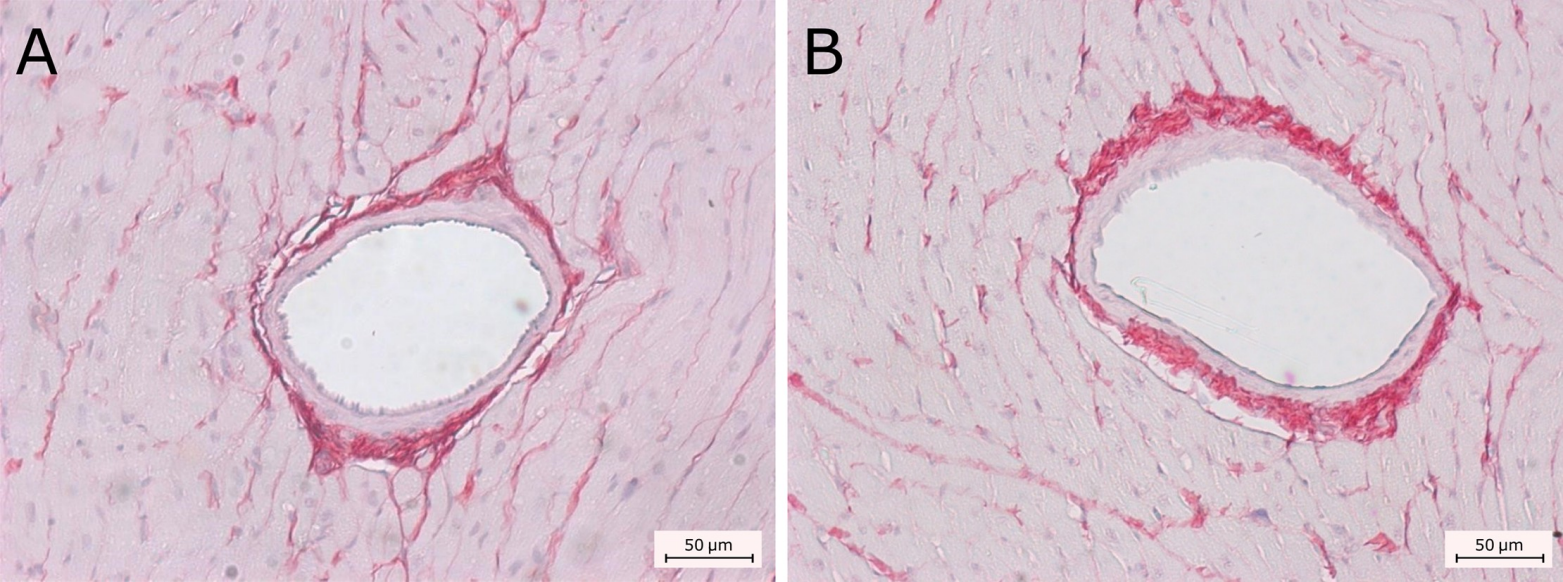
Determination of ventricular perivascular fibrosis. Ventricular perivascular fibrosis was analyzed in the myocardium of control (A) and diabetic animals (B). No differences were observed (*P* = 0.36) between groups.

Atria presented higher values of interstitial collagen when compared to ventricles in diabetic animals (*P* = 0.01) and control animals (*P* = 0.03). Representative images perivascular collagen from ventricular arterioles stained with picrosirius red are shown in Fig 5.

## Discussion

Diabetic cardiomyopathy is characterized by structural, metabolic and functional changes in the myocardium in the absence of other cardiac risk factors, such as ischemia, hypertension or other pathophysiological processes that may justify heart failure. DCM is usually asymptomatic in the early stages of its evolution. In the clinical setting, it is initially seen as diastolic dysfunction with LV hypertrophy and decreased LV relaxation [7].

In this paper we used the STZ‐injection type 1 DM model (insulin insufficiency), which is known for its effects on myocardial mechanics [13] and is considered a model for DCM [11]. Injection of STZ resulted in a diabetic state characterized by hyperglycemia and loss of body weight in agreement with the literature [12].

We studied cardiac mechanics at the cellular level by measuring contraction and relaxation in LV isolated cardiac myocytes. We observed changes in both contraction and relaxation, including an increase in the time to reach maximal shortening velocity (TMS), time to peak of contraction (TPC), time to maximal relaxation velocity (TMR) and time to 50% resting sarcomere length (THALF). Changes in contractility in single myocytes in STZ diabetes (6 to 12-week) in rats have been reported [21, 22]. Alterations in cell shortening in response to stimulation have been reported after short term STZ-induced diabetes using confocal microscopy and morphometric parameters [34]. Diastolic dysfunction is an early manifestation in DCM and diabetic patients with normal myocardial collagen content and ejection fraction were shown to have increased isolated cardiomyocyte resting tension [35].

Calcium handling disturbances appears to play a major role in changes observed in isolated myocytes from diabetic animals, involving prolonged SERCA mediated Ca^2+^ removal during diastole and changes in myofilament Ca^2+^ sensitivity [36]. The increased time constants we observed in isolated cardiomyocytes are in agreement with the literature consensus about the involvement of [Ca^2+^]_*I*_ cycling alteration during T1DM, with studies reporting changes in calcium transients and SERCA function and expression [37]. The prolonged [Ca^2+^] transient decay is a result of sarcoplasmic reticulum reuptake impairment during the relaxation phase leading to slower relaxation and diastolic dysfunction [38].

*In vivo* cardiac function was assessed in diabetic and control animals by ECG and high-resolution echocardiographic imaging. Echocardiographic findings showed reduced systolic peak velocity (S’) pointing to reduced segmental longitudinal systolic function accompanied by changes in cardiac morphology, with increased LA dimension in diabetic compared to control rats. Increase in LA diameter may be related to increased LV stiffness and end-diastolic pressure.

Alterations in atrial conduction with prolonged action potential were first observed in electrically stimulated isolated atria after 1 week of STZ injection in rats [39]. Increased LV action potential duration was demonstrated in vivo in STZ diabetic rats 10 weeks after induction [40] and in Langendorff perfused rat hearts (16 weeks) [41]. The prolonged P-wave in diabetic rats observed in our paper may reflect atrial conduction disturbances. In patients, P wave duration has been suggested as an index for left ventricular diastolic dysfunction [42].

It is of note that the wider P-wave observed in our study in diabetic animals was concomitant to an increase LA dimension [43]. Further investigations are necessary to elucidate this finding and its relevance in DCM. In children with T1DM, P wave dispersion was higher compared to controls [44].

In our study diabetic rats showed an increase in plasma levels of hepatic aspartate aminotransferase and alkaline phosphatase, which may suggest hepatic damage induced by diabetes [45].

We found increased levels of plasma triglycerides (Table 4) in diabetic rats in this early phase of the disease, similar to what we have reported for the same experimental model at 4 weeks after injection were we found a concomitant increased in TG in the LV [46]. These changes are likely related to the adapted mode of operation induced in the heart during hypoinsulinemia, with decreased glycolysis and ATP levels, which might enhance ATP/ADP-AMP cycling.

We observed no differences in collagen content in the interstitium and in the perivascular area of diabetic animals when compared to controls. This suggests that, at such an early stage, the observed changes in contractility may not involve increase in wall stiffness from collagen deposition, as is usually reported in later periods of this animal model of T1DM. The presence of interstitial fibrosis and perivascular myocardial fibrosis is a common finding after 8 weeks of STZ injection and it is usually considered an important factor in the reduced contractility seen in the later stages [47].

In one of the few studies that address early stages of the T1DM STZ-model, Becher et *al*. [48] found reduced LV contractility, LV relaxation and increased stiffness at 2 weeks after STZ injection. They made direct hemodynamic measurements with a conductance catheter technique. Differently from our findings, they found increased picrosirius red staining after 2 weeks of STZ injection.

We found significant decreases in both contractile and relaxation parameters in the isolated cardiomyocytes from diabetic animals, which are the most relevant functional alterations observed at this stage. Impairment of electrical conductivity from changes in interstitial collagen are not likely a relevant event at this stage as well. However, the observed increase in left atrial dimension may be associated to the appearance of other electrical disturbances.

One limitation of this experimental model, especially if results are to be applied to therapeutic strategies, is the short duration of the diabetic state which is substantially different than the slowly and accumulating damage of diabetes in humans. T1DM is a chronic disease that develops over years even with strict glycemic control and insulin therapy. Another relevant limitation is the lack of comparison with other time-points in the same animals, which would further corroborate our findings.

In conclusion, the present study demonstrates reduced systolic function with impaired myocyte contractility in early-stage type-1 diabetic rats, which is attributed to the effects of diabetes at the cardiomyocyte level. The data suggest that alterations in longitudinal systolic heart function observed in diabetic animals are associated to the prolonged time course of shortening and relaxation of isolated ventricular myocyte. The changes observed in isolated myocytes may represent the initial cellular substrate for diabetic cardiomyopathy, already manifested in an early phase of DM in this experimental model. We found early functional changes, including contraction abnormalities in isolated cells, systolic alterations in vivo and changes in duration and variability of P wave in ECG, occurring before signs of interstitial and ventricular perivascular fibrosis. This may indicate that the present STZ model may be suitable to investigate the initial phase of DCM, the pathophysiological mechanisms involved and possible effects of drug interventions.
